# Comparisons between ankle distraction arthroplasty and supramalleolar osteotomy for treatment of post-traumatic varus ankle osteoarthritis

**DOI:** 10.1186/s12893-022-01623-x

**Published:** 2022-05-14

**Authors:** Zongyu Yang, Liang Cui, Shiwu Tao, Jianyong Zhao, Li Wang, Fengqi Zhang, Xinzhong Shao

**Affiliations:** 1 Department of Orthopaedic Surgery, The Third Hospital of Hebei Medical University, Hebei 050051 Shijiazhuang, People’s Republic of China; 2 Department of Sports Medicine-Foot and Ankle Surgery, Cangzhou Hospital of Integrated TCM-WM, Hebei 061001 Cangzhou, People’s Republic of China; 3 Department of Hand Surgery, The 3rd Hospital, Hebei Medical University, No. 139 Ziqiang Road, Shijiazhuang, 050051 People’s Republic of China

**Keywords:** Post-traumatic ankle osteoarthritis, Clinical outcome, Ankle distraction arthroplasty, Supramalleolar osteotomy, Comparative study

## Abstract

**Background:**

Ankle distraction arthroplasty and supramalleolar osteotomy were both options for post-traumatic varus ankle arthritis (VAA), but their comparative effectiveness was scarcely reported. This study aimed to compare the outcomes of two operative methods for treatment of Takakura-Tanaka stage 3 post-traumatic VAA.

**Methods:**

This was a retrospective study, comprising 73 consecutive patients who presented with Takakura-Tanaka stage 3 post-traumatic VAA treated by either ankle distraction arthroplasty (n = 32) or supramalleolar osteotomy (n = 41) from January 2016 to December 2019. All patients had a minimum 24-month follow-up assessments. The outcome measures were visual analog scale (VAS), the American Orthopedic Foot & Ankle Society (AOFAS) ankle-hindfoot scores, complications, patient-rated overall satisfaction and ankle function.

**Results:**

At an average of 32 months (range, 24–52 months) follow-up, significant improvement was observed for VAS, AOFAS, range of motion (ROM) and most radiographic parameters (except for TAS and TLS for ankle distraction arthroplasty group) compared to preoperative baselines (*p* < 0.05) for both groups. However, both groups did not differ significantly in terms of VAS or AOFAS, excellent and good rate (78.1% versus 85.4%, p = 0.422), overall rate of postoperative complications (28.1% vs. 17.1%, p = 0.257), or various radiographic parameters (e.g. tibial anterior surface angle, talar tilt angle and tibial lateral surface angle) (all p > 0.05). The ankle distraction arthroplasty group had a better postoperative ankle motion than did the supramalleolar osteotomy group, in terms of plantarflexion (37.8 ± 4.2 vs. 30.4 ± 3.6, *p* = 0.006), dorsiflexion (36.5 ± 6.4 vs. 28.3 ± 5.5, *p* = 0.004), varus (32.1 ± 4.5 vs. 27.1 ± 3.1, *p* = 0.017) and valgus (28.4 ± 3.7 vs. 25.2 ± 2.8, *p* = 0.046).

**Conclusions:**

Both operative treatments are effective for Takakura-Tanaka stage 3 post-traumatic VAA. In practice, individualized treatment option tailored to the ankle condition and patients’ specific need should be considered.

*Level of evidence*: III, retrospective comparative series.

## Introduction

Ankle osteoarthritis is an important cause of ankle pain and functional limitation, despite the lower incidence rate than knee and hip osteoarthritis [1]. Anatomically, ankle joint had approximately 1/2–3/5 the maximum cartilage thickness at weight-bearing areas as that of hip and knee joint (2.7 mm vs. 3–6 mm) [2]. Thus, any abnormal alignment of the ankle would lead to local stress concentration and accelerate the degeneration of ankle cartilage and the development of osteoarthritis [3]. It was estimated that 20–40% of patients with acute ankle trauma like ankle sprain would develop chronic sprain, leading to injury or even rupture of the lateral ligaments, causing chronic lateral ankle instability and the secondary post-traumatic varus ankle osteoarthritis (VAA) [4]. Recent reports showed that the young patients were increasingly the predominant for this arthropathy and the incidence seemed on the rise [5, 6].

Currently, for end-stage post-traumatic VAA (stage 4 classified by Takakura-Tanaka classification), ankle arthrodesis was considered as the standard treatment [7, 8]. Also, for stage 1 and 2 VAA, arthroscopic surgery or open joint debridement is considered as the standard treatment, accepted by the majority of patients and the reported clinical outcome are favorable. However, for stage 3 post-traumatic VAA, there is lack of substantial evidences regarding which surgical treatment method is optimal. The most commonly used surgical options are ankle distraction arthroplasty and supramalleolar osteotomy, but no overwhelming consensus has been reached to support either method, because each had respective advantages and disadvantages [1, 2, 9]. The ankle distraction arthroplasty could achieve the early functional recovery, but was associated with relatively high failure rate, especially for those obese patients and those with large talar tile angles [2, 9]; in contract, supramalleolar osteotomy was advantageous in correcting the load-bearing line of the ankle and hindfoot and has fewer complications, but was limited by the inability to achieve a fast recovery [1]. To our best knowledge, there are no studies that directly compared the outcomes of both surgical methods for stage 3 VAA.

Considering the critical importance of evidence-based data on decision-making for the surgical option, it is necessary to conduct a study that directly compares the outcomes between both surgical treatments. Thus, we conducted this study, with aims to compare the clinical outcomes between ankle distraction arthroplasty and supramalleolar osteotomy for stage 3 VAA, in terms of pain relief, functional recovery and postoperative complications.

## Methods

This was a retrospective study. The study protocol was in accordance with the Helsinki Declaration and was approved by the institutional review board of Cangzhou Hospital of Integrated TCM-WM and all patients provided the written informed consent.

From January 2016 to December 2019, consecutive patients who were diagnosed with post-traumatic VAA were potentially eligible for study. The inclusion criteria were: age 18 years or older; stage 3 post-traumatic VAA classified as Takakura-Tanaka classification; ankle pain and swelling lasting > 3 months not responsive to conservative treatment; absence of past any surgical procedure around the ankle joint; complete pre- and postoperative data and imaging examinations, and a minimum 24-month assessment. The exclusion criteria were recent infection around the ankle joint; other serious deformities or diseases of the foot and ankle, such as clubfoot or diabetic foot; incomplete medical record documentations or loss to follow-up or follow-up period < 24 months. Additionally, surgery is contraindicated for those with congenital collagen deficiency, bodyweight > 120 kg, severe heart disease, lesions affecting liver and kidney function, severe diabetes, central nervous system diseases or others.

### Preoperative evaluation

Preoperative evaluation included a detailed history of ankle osteoarthritis, comorbidities, physical examination, and imaging examination. On the anteroposterior view of ankle radiographs, tibial anterior surface angle (TAS), talar tilt angle (TT) and tibial lateral surface angle (TLS) were measured. The calcaneal axial radiograph was taken to assess the force line of the lower limb and evaluate the presence of varus or valgus of the calcaneus and talus; CT scans were performed to evaluate the condition of the subtalar and tibiotalar joint; MRI was performed to evaluate the cartilage condition, presence or absence of talar necrosis, surrounding soft tissue, edema of the surrounding ligaments, and completeness of the lateral ligaments.

### Operative procedures

#### Supramalleolar osteotomy

The patient was placed in supine position and surgery was performed under lumbar or/and epidural anesthesia with a thigh tourniquet control. Prophylactic intravenous antibiotics (generally, third-generation cephalosporin) was administered 30 min prior to skin incision. A 4-cm longitudinal incision was made in the middle of the anterior ankle to expose the ankle joint cavity to determine the presence or absence of tibiotalar impingement. Intraoperatively, lip-like hyperplastic tissues of the tibial articular surface and the lateral talar articular surface can be observed, with limited passive movement of the ankle. The hyperplastic osteophytes were excised, and the ankle joint was passively moved until the normal range of motion (ROM) was reached. The joint cavity was washed with normal saline and the surgical incision was sutured. At 4–5 cm above the ankle joint, a guiding Kirschner wire was used for osteotomy and osteotomy direction was confirmed the under fluoroscopy; then, the medial, anterior, and posterior bone cortices were cut from medial to lateral, parallel to the tibial articular surface, and the osteotomy gap was then opened with an osteotomy spreader and filled with allogeneic bone to increase the stability; the contralateral bone cortices and periosteum were retained to form a hinge. Satisfactory correction of the varus deformity was confirmed by fluoroscopy, a Kirschner wire was used for temporary fixation, and anatomical plate was used for rigid fixation. The incision was carefully sutured and dressed.

Postoperatively, routine dressing changes were performed and prophylactic intravenous antibiotics were administered. From postoperative day 1, patients were instructed to exercises the ipsilateral toes and quadriceps femoris to prevent lower-extremities deep venous thrombosis. The ankle was half loaded by 1 month postoperatively, and full loaded by 2 months postoperatively.

**Fig. 1:**
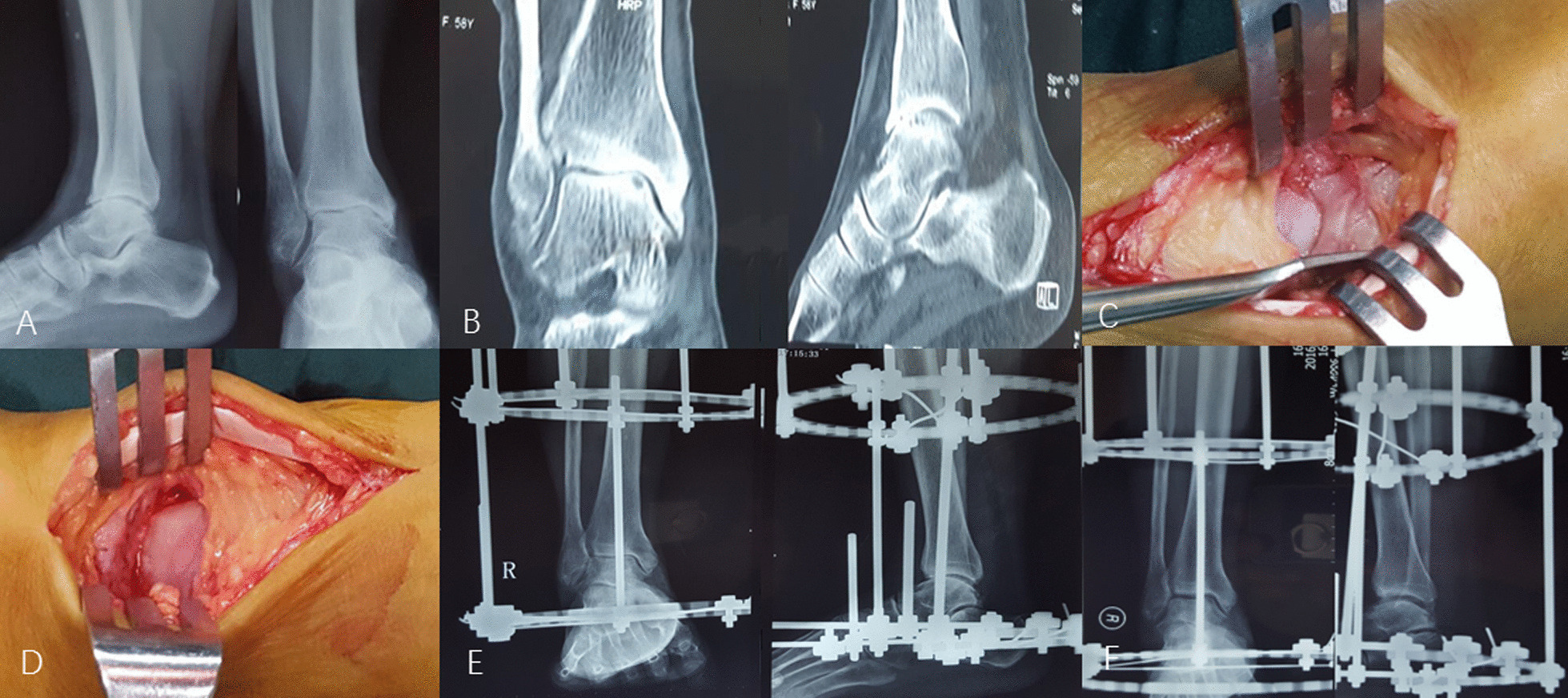
Depicts a female, 58 years, who had right post-traumatic varus ankle osteoarthritis classified as Takakura-Tanaka stage 3 and underwent ankle distraction arthroplasty and got a favorable outcome. The preoperative radiographs (**A**, lateral and anteroposterior view) and CT scans (**B**) narrow articular space and osteophytes, and the slight varus deformity. The osteophytes were seen and removed (**C**, **D**). seven days after operation, the ankle articular space was slightly widened (**E**). Radiographs taken at 1.5 months after operation showed marked improvement of ankle articular space, almost to normal (**F**)

Figure 1 depicts a typical case treated by Supramalleolar osteotomy.

#### Ankle distraction arthroplasty

The same preoperative preparation and debridement procedure was implemented, the same as the supramalleolar osteotomy technique. The ankle joint was placed in a neutral position and the annular external fixator was placed in an appropriate position with the extension rod directly opposite the ankle joint activity center. A 2.0-mm-diameter Kirschner wire was used to drill approximately 8 cm below the knee joint, and then two Kirschner wires were drilled approximately 5 cm above the ankle joint, parallel to the knee joint; one Kirschner wire was fixed in front of the calcaneal tubercle, and the other one was fixed in the metatarsal base of the anterior foot. Each ring was reinforced with a threaded needle.

Postoperatively, the same incision management regimen was implemented. The external fixator was adjusted to stretch the ankle joint cavity by about 0.5 mm every day, once every 12 h, until the ankle joint space was to 5 mm. The ankle was half loaded by 1 month postoperatively, and full loaded by 2 months postoperatively. At 3 months postoperatively, the external fixator was removed and ankle rehabilitation training was commenced. The supramalleolar osteotomy group performed the same functional exercises to prevent postoperative ankle stiffness and enhance the joint ROM.

**Fig. 2:**
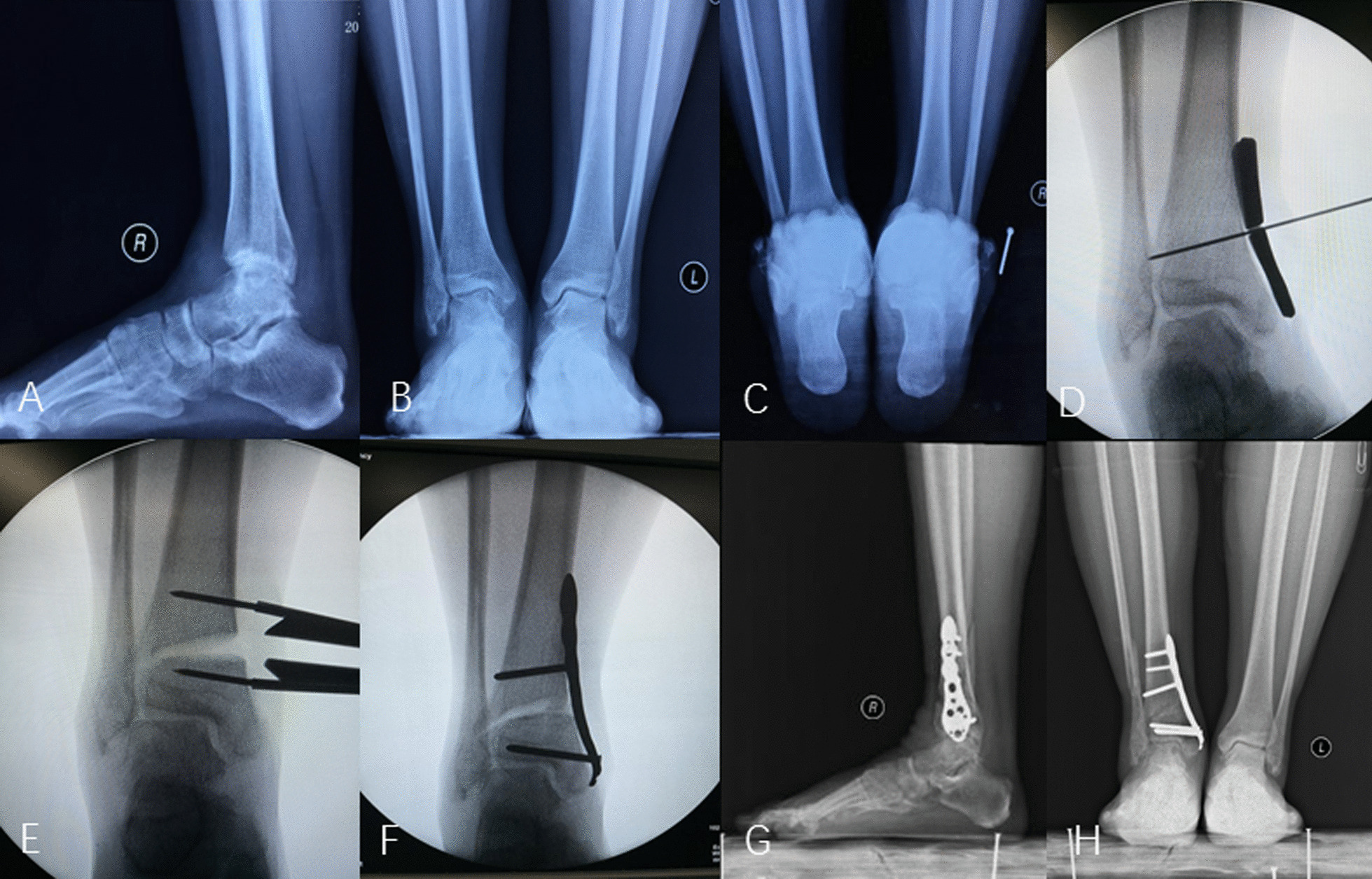
Depicts a female, 48 years, who had right Takakura-Tanaka stage 3 varus ankle osteoarthritis and underwent supramalleolar osteotomy to reconstruct the mechanical axis. Preoperative radiographs **A**–**C** (lateral, anteroposterior, and calcaneal axial view) showed the ankle varus deformity and osteoarthritis; **D**–**F** showed the operative procedure process (localization of supramalleolar osteotomy, widening the gap, and internal fixation). **G**, **H** Showed the improved force line of the ankle joint and the articular space

Figure 2 depicts a typical case treated by ankle distraction arthroplasty.

#### Outcome measures

Postoperatively, patients were routinely followed up at outpatient at 1 month, 3, 6, 12 and 24 months, and afterwards as needed. At each visit, anteroposterior and lateral radiographs of ankle joint and full-length lower extremity weight-bearing radiographs were taken. A goniometer was used to measure the TAS, TT, and TLS.

All patients were assessed by the investigators (Z Yang and LWang) preoperatively and at each outpatient visit.

The ROM of the operated ankle was measured, including varus, valgus, dorsiflexion, and plantarflexion. The American Orthopedic Foot and Ankle Society (AOFAS) ankle-hindfoot score was used to evaluate the pain, ankle function, gait, and force line of the affected ankle. Visual analog scales (VAS) scores for used to evaluate pain. At the last visit, patients were asked to rate their overall satisfaction with their surgical results as excellent, good, fair, or poor. (Table 1)

**Table 1:** Clinical rating scale for postoperative ankle function

Rating	Description
Excellent	Full range of motion equal to the contralateral ankle without pain. Un-restricted work or sports activity
Good	Functional range of motion and stable ankle. Able to return to the previous level with minimal pain with work or sport activity
Fair	Functional range of motion, good stability, moderate level of pain, and/or stiffness with activities of daily living and sports activity
Poor	Persistent instability or pain, the same or worse than before surgery

During the entire postoperative period, operation or hardware related complications would be documented in the hospitalization medical records or the follow-up register at each outpatient visit.

### Statistical analyses

Data collected at the last visit were used for comparisons with the preoperative baseline parameters, namely the in-group comparisons; also, between-group comparisons between two treatments at the last visit were performed. Continuous data were presented with mean ± standard deviation (SD) and their normality status was detected by Kolmogorov-Smirnov test, whereby Student-*t* test or Mann Whitney-*U* test was used for between-group comparisons, paired Student-t test for in-group comparisons. Categorical data were presented with number and percentages, and Chi-square or Fisher’s exact test was used for between-group comparisons.

*p* < 0.05 was considered as statistically significant for all analyses. SPSS 25.0 (IBM, Armonk, New York, USA) was used to perform all analyses.

## Results

There were 73 consecutive patients eligible for inclusion, including 32 in ankle distraction arthroplasty group and 41 in supramalleolar osteotomy group. Among ankle distraction arthroplasty group, there were 19 men and 13 women, with an average age of 54.7 ± 12.8 years; left ankle was affected in 14 patients and right in 18. Among supramalleolar osteotomy group, 27 were men and 14 were women, with an average of 56.4 ± 11.7 years; left ankle was affected in 17 patients and right in 24. No significant differences were observed between the two groups for any baseline parameter (Table 2). The operative procedure lasted 78 ± 32 min for ankle distraction arthroplasty, significantly longer that did the supramalleolar osteotomy (94 ± 28 min).

**Table 2:** Comparison of the data of patients with ankle distraction arthroplasty and supramalleolar osteotomy

Variable	Ankle distraction arthroplasty (n and %, or mean ± SD)	Supramalleolar osteotomy (n and %, or mean ± SD)	*p*
Age	54.7 ± 12.8	56.4 ± 11.7	0.801
Gender			0.569
Male	19 (59.4)	27 (65.9)	
Female	13 (40.6)	14 (34.1)	
Side			0.402
Left	14 (43.8)	17 (34.1)	
Right	18 (56.3)	24 (65.9)	
Body mass index (kg/m^2^)	27.3 ± 3.4	27.7 ± 4.3	0.762
Time interval between onset and operation (years)	3.2 ± 4.3	4.1 ± 3.5	0.244
Follow up period (month)	30.8 ± 5.5	32.9 ± 7.4	0.601

The average follow-up period was 32 months (range, 24–52 months). The final assessments showed significant improvement, compared to preoperative baseline parameters, for almost all the variables for both groups (*p* < 0.05), except for TAS (*p* = 0.376) and TLS (*p* = 0.179) in ankle distraction arthroplasty group (Table 3).

**Table 3:** Between-group and within-group comparison of clinical and radiographic outcome measurements for ankle distraction arthroplasty and supramalleolar osteotomy

	Ankle distraction arthroplasty (n = 32)			Supramalleolar osteotomy (n = 41)			Between-group comparison at last visit
	Preoperative	Postoperative	^*^p	Preoperative	Postoperative	^*^p	^***#***^ ***p***
AOFAS score	47.1 ± 8.9	84.6 ± 6.4	< 0.001	45.4 ± 5.7	86.5 ± 7.2	< 0.001	0.564
VAS	5.6 ± 1.4	1.4 ± 1.3	0.001	5.8 ± 1.2	1.1 ± 1.4	< 0.001	0.163
TAS (°)	84.7 ± 5.7	86.2 ± 4.1	0.376	80.2 ± 4.6	92.1 ± 3.9	< 0.001	0.002
TT (°)	4.5 ± 2.1	3.2 ± 1.3	0.014	6.3 ± 3.4	2.8 ± 2.0	< 0.001	0.279
TLS (°)	78.4 ± 3.9	81.2 ± 2.5	0.179	76.2 ± 5.8	82.1 ± 6.5	0.008	0.768
Range of motion (ROM)							
Plantarflexion (°)	23.3 ± 3.7	37.8 ± 4.2	< 0.001	25.1 ± 4.8	30.4 ± 3.6	0.015	0.006
Dorsiflexion (°)	17.5 ± 5.8	36.5 ± 6.4	< 0.001	23.8 ± 6.1	28.3 ± 5.5	0.043	0.004
Varus (°)	23.6 ± 6.0	32.1 ± 4.5	0.007	22.7 ± 4.2	27.1 ± 3.1	0.039	0.017
Valgus (°)	19.8 ± 4.1	28.4 ± 3.7	0.006	20.0 ± 3.4	25.2 ± 2.8	0.011	0.046

^*^Within-group comparison between preoperative and postoperative outcome measurement; ^#^ between-group comparison at the last visit

At the last visit, the ankle distraction arthroplasty group had significantly better ankle mobility range in terms of varus, valgus, dorsiflexion, and plantarflexion than did the supramalleolar osteotomy group (***p*** values, 0.006 to 0.046) (Table 3). There were no significant differences as for TT (2.8 ± 2.0 vs. 3.2 ± 1.3, ***p*** = 0.279) and TLS (82.1 ± 6.5 vs. 81.2 ± 2.5, ***p*** = 0.768), but a significant higher TAS was found in the supramalleolar osteotomy group (92.1 ± 3.9 vs. 86.2 ± 4.1, ***p*** = 0.002) (Table 3). The ankle distraction arthroplasty had a similar mean AOFAS score (84.6 ± 6.4) and VAS pain score (1.4 ± 1.3) as the supramalleolar osteotomy group (AOFAS score, 86.5 ± 7.2; VAS pain score, 1.1 ± 1.4). The mean AOFAS and VAS pain scores were significantly improved postoperatively compared with preoperatively in both groups (both P < 0.001). (Table 3)

No significant difference for complication prevalence rate was found between both groups (28.1% vs. 17.1%, *p* = 0.257). There were nine complications in the ankle distraction arthroplasty group, including sinus infection of the Kirschner wire and exudation of secretions in four patients, persistent chronic ankle pain in one patient who finally underwent ankle arthrodesis at 31 months after the index operation, readjustment of the external fixator due to an accident in one patient, and ankle stiffness in three patients who, afterwards, improved the ankle motion substantially via reinforced rehabilitation training. There were seven complications in the supramalleolar osteotomy group, including 3 cases of scar contracture, 2 cases of superficial surgical incision which later resolved by oral antibiotics, and 2 cases of delayed healing at the osteotomy site which were treated by extracorporeal shock wave and resolved.

Among ankle distraction arthroplasty group, the self-reported satisfaction was excellent by 15 (46.9%) patients, good by 10 (31.3%), fair by 5 (15.6%), and poor by 2 (6.3%). While among supramalleolar osteotomy group, the self-reported satisfaction was excellent by 20 (48.8%) patients, good by 15 (36.6%), fair by 4 (9.8%) and poor by 2 (4.9%). The excellent and good rate was not significantly different between two groups (78.1% versus 85.4%, p = 0.422) (Table 4).

**Table 4:** Self-reported satisfaction by patients between ankle distraction arthroplasty and supramalleolar osteotomy group

	Excellent	Good	Fair	Poor	^*&*^*p*
Ankle distraction arthroplasty	15	10	5	2	0.422
Supramalleolar osteotomy	20	15	4	2	

^&^Comparison between both groups for the excellent and good rate (78.2% vs. 85.4%, p = 0.422)

## Discussion

The present study retrospectively analyzed 73 patients with Takakura-Tanaka stage 3 post-traumatic VAA treated with either ankle distraction arthroplasty or supramalleolar osteotomy. The results suggest that ankle distraction arthroplasty is advantageous in restoring ankle motion range, while supramalleolar osteotomy perform better in correcting TAS. However, in terms of others, e.g. AOFAS, VAS, TT, TLS, patients’ self-reported satisfaction, and overall complication rate, both treatments did not differ.

Ankle distraction arthroplasty was advocated by Valburg et al. [15] in 1995 to treat post-traumatic ankle osteoarthritis, and the preliminary study of 11 patients showed significant improvement in pain, mobility and joint space. The mechanism was that the abnormal mechanical stress of the ankle was reduced via distraction, whereby flow of synovial fluid in the joint was intermittently promoted, creating an improved circumstance for repair of articular cartilage. In the later studies, this procedure proved to be effective in relieving pain and restoring the ankle function [16–20]. However, the relatively low efficiency rate, inconvenience and need of longer period of treatment may limit its more extensive use in practice [21]. Additionally, the gradually lowering postoperative satisfaction over time should be a concern and for patients with obvious ankle valgus deformity, ankle joint distraction arthroplasty alone cannot correct the deformity [22].

The superiority of supramalleolar osteotomy over distraction arthroplasty was the ability to correct the load line of the ankle and hindfoot and to correct the distal tibial deformity in the coronal and sagittal planes [1, 23]. In this study, supramalleolar osteotomy proved to better correct the talus varus deformity and restore the lower limb alignment, consistent with the previous findings [24, 25], thus delaying the development of ankle osteoarthritis. It should be noted that, for patients with chronic ankle instability caused by severe injury or repeated multiple injuries who may develop increased stress in the asymmetric joint spaces [2], additional osteotomy procedure was generally needed to restore the lateral stability [26].

The relatively lower rate of postoperative complications might also be an advantage for the supramalleolar osteotomy, despite that we did not observe the significant difference (17.1% vs. 28.1%). This non-significant difference was likely caused by the small sample size, 32 participants in ankle distraction arthroplasty group and 41 in supramalleolar osteotomy group. The previous studies reported the similar complication rate as ours and complications such as bone nonunion at the osteotomy site was also sporadically reported [27, 28]. Despite that, supramalleolar osteotomy was not strongly recommended, largely due to the need for a second operation to remove the hardware and the higher demand of ankle mobility, especially for the young patients.

In the present study, we did not find the significant difference in overall satisfaction rate, but a tendency towards lower value in those treated by ankle distraction arthroplasty (excellent and good rate of 78.1% versus 85.4% for supramalleolar osteotomy). This may be caused by the higher rate of complications associated with ankle distraction arthroplasty, including sinus tract infection, fixation failure, difficulty in moving after surgery, and the need for frequent reviews and external fixation adjustments [16].

This study suffered from several limitations. First, the retrospective design might have impeded the accuracy and precise in data collection. The setting of specific investigators responsible for measuring the parameters would partly compensate for this limitation. Second, due to the limited use in our institution, only 73 eligible patients were included for data analysis, making the comparison not definitely conclusive. It was possible the true differences between two treatments for some outcome variables were hampered by limited statistical power caused by small sample size, which was known as type II statistical error. Third, these operative procedures were performed by orthopaedic surgeons (n = 7) and foot and ankle surgeons (n = 4), and their experience might have affected the results. But it is a pity that we could not compare this confounding effect because of the very limited operated cases for one surgeon. Fourth, the single-center design would have lowered the generalizability of our results to other settings.

## Conclusions

Both ankle distraction arthroplasty and supramalleolar osteotomy are effective treatment methods for Takakura-Tanaka stage 3 post-traumatic VAA. Ankle distraction arthroplasty was advantageous in restoring ankle mobility, while supramalleolar osteotomy performed better in correcting ankle varus deformity and had a trend towards fewer complications. The results should be verified by the higher-level-evidence studies. In practice, individually tailored treatment option, based on the ankle conditions and patients’ specific need, should be considered.

## Data Availability

All the data will be available upon motivated request to the corresponding author of the present paper.
